# Time Series Gene Expression Profiling and Temporal Regulatory Pathway Analysis of Angiotensin II Induced Atrial Fibrillation in Mice

**DOI:** 10.3389/fphys.2019.00597

**Published:** 2019-05-29

**Authors:** Yu-Xuan Wu, Xiao Han, Chen Chen, Lei-Xin Zou, Zhi-Chao Dong, Yun-Long Zhang, Hui-Hua Li

**Affiliations:** ^1^Department of Cardiology, Institute of Cardiovascular Diseases, First Affiliated Hospital of Dalian Medical University, Dalian, China; ^2^Department of Nutrition and Food Hygiene, School of Public Health, Dalian Medical University, Dalian, China

**Keywords:** angiotensin II, atrial fibrillation, microarray, time series gene expression profiling, Pik3cg

## Abstract

**Background/Aim:** Angiotensin II (Ang II) and hypertension play critical roles in the pathogenesis of the atrial remodeling that contributes to atrial fibrillation (AF). However, the gene expression profiles and signaling pathways in atria during the development of AF induced by Ang II remain unknown.

**Methods:** Wild-type male mice (C57BL/6 background, 10 weeks old) were administered an infusion of Ang II (2000 ng/kg/min) using an osmotic pump for 1, 2, and 3 weeks. Blood pressure (BP) was measured by the tail-cuff method. AF was induced and recorded. Atrial enlargement and remodeling were examined by echocardiography and Masson’s trichrome staining. Time-series microarray analyses were conducted to examine gene expression profiles and pathways.

**Results:** Ang II infusion resulted in marked elevation of systolic BP, increased AF incidence and duration, atrial enlargement, fibrosis, and atrial infiltration of myofibroblasts and F4/80-positive macrophages in a time-dependent manner. Microarray results showed that 1,719 genes were differentially expressed in the atrium at weeks 1, 2, and 3 after Ang II infusion. Gene ontology showed that these genes participate mainly in immune system processes, and regulation of cell migration, cell adhesion, complement activation, and the inflammatory response. Significant pathways included lysosomal and phagosomal pathways, which are involved in antigen processing and presentation, as well as chemokine signaling, and extracellular matrix–receptor interaction, which are known to play important roles in Ang II-induced AF. Moreover, these differentially expressed genes were classified into 50 profiles by hierarchical cluster analysis. Of these, eight profiles were significant and contained a total of 1,157 genes. Gene co-expression network analysis identified that Pik3cg (also known as phosphoinositide-3-kinase regulatory subunit 3) was localized in the core of the gene network, and was the most highly expressed among the Pik3 isoforms at different time points.

**Conclusion:** The present findings revealed that many genes are involved in Ang II-induced AF, and highlighted that Pik3cg may play a central role in this disease.

## Introduction

Atrial fibrillation (AF) is the most prevalent cardiac arrhythmia in humans, and is associated with an increased risk of stroke, heart failure (HF), and death (Gao and Dudley, 2009). AF is characterized by structural and electrical remodeling, which frequently results in the recurrence and maintenance of arrhythmia (Nattel et al., 2007; Gao and Dudley, 2009). The mechanisms underlying the development of AF are complex; however, the renin–angiotensin system (RAS) plays a critical role in the development of AF (Griendling et al., 2000; Kumagai et al., 2003; Gao and Dudley, 2009; Gramley et al., 2010). Angiotensin II (Ang II) is the major effector hormone of the RAS, which exerts a key role in regulation of inflammatory response, oxidative stress and atrial fibrosis during AF (Cohn et al., 2000; Gao and Dudley, 2009). Moreover, increased levels of Ang II induce elevation of blood pressure (BP) and hypertrophic remodeling, eventually resulting in, whereas blocking of RAS markedly attenuates and reverses these diseases (Cohn et al., 2000; Clarke et al., 2013).

Increasing evidence from animal models and human studies have revealed that multiple signaling pathways, including NF-kB, NADPH oxidase, and TGF-β/Smad, are involved in the development of AF (Griendling et al., 2000; Kumagai et al., 2003; Gao and Dudley, 2009; Gramley et al., 2010). Furthermore, human studies have identified that alterations of 10 novel genetic loci, including METTL11B/KIFAP3, ANXA4/GMCL1, CEP68, TTN/TTN-AS1, KCNN2, KLHL3/WNT8A/FAM13B, SLC35F1/PLN, ASAH1/PCM1, SH3PXD2A, and KCNJ5, are associated with AF susceptibility (Christophersen et al., 2017). However, the molecular mechanism of how Ang II causes the initiation and development of AF remains to be elucidated. In this study, we analyzed the gene expression profiles of the atrium from Ang II-induced wild-type (WT) mice using time-series microarray assays.

## Materials and Methods

### Animal Models

Male C57BL/6J mice (8–10 weeks-old, *n* = 10 per group) were infused with angiotensin II (Ang II, 2000 ng/kg/min) via subcutaneously implanted osmotic minipumps (Model 1007D; Alzet, Cupertino, CA, United States) for 1, 2, and 3 weeks (Verheule et al., 2004; Purohit et al., 2013). There was no any difference in mortality, wound infection or wound healing after Ang II infusion in models compared to saline-treated mice. Systolic BP was measured by the tail-cuff method before starting treatment and every 2 days after Ang II infusion as described (Zhang et al., 2014; Wang et al., 2016). Animals were anesthetized with isoflurane (1.5%) and then underwent M-mode echocardiography at each time point using a 30 MHz probe (Vevo 1100 system, Visual Sonics, Toronto, ON, Canada) (Wang et al., 2016; Li et al., 2018). All procedures were approved by and performed in accordance with the Animal Care and Use Committee of Dalian Medical University. This study conformed to the Guide for the Care and Use of Laboratory Animals published by the U.S. National Institutes of Health.

### Induction of Atrial Fibrillation

At the end of Ang II infusion, mice from all four groups (*n* = 10 per group) were anesthetized with 2.5% tribromoethanol (0.02 mL/g; Sigma-Aldrich, United Kingdom). Animal body temperature was controlled at 37.0 ± 0.5°C using mouse pad circuit board equipped with a heating element (Nomoypet, JRD-7w, China). Intracardiac pacing was performed by inserting an eight-electrode catheter (1.1 F, octapolar EP catheter, Science) through the jugular vein and advancing it into the right atrium and ventricle (Abdulghani et al., 2014). Atrial arrhythmias were introduced by applying 5-s bursts through the catheter electrodes using the automated stimulator that was part of the data acquisition system. The cycle length (CL) in the first 5-s burst is 40 ms and decreases in each successive burst with a 2-ms decrement down to a CL of 20 ms according to our previous report (Luo et al., 2013; Li et al., 2018). A computer-based data acquisition system was used to record a 1-lead body surface ECG and up to 4 intracardiac bipolar electrograms (GY6328B; HeNan HuaNan Medical Science and Technology, Ltd.).

### Histological Examination

The left atrium from each mouse (*n* = 8 per group) was fixed in 4% paraformaldehyde and for 24 h, embedded in paraffin and sectioned. The section (5 μm) was with Masson’s trichrome and immunohistochemistry as described (Li et al., 2018). The images were evaluated by a pathologist in a double-blinded manner, and were analyzed using a Nikon microscope (Tokyo, Japan).

### Microarray Assay and Bioinformatics Analysis

All mice were euthanized by an overdose of pentobarbital sodium (100 mg/kg) administered via intraperitoneal injection at weeks 1, 2, and 3 post Ang II infusion. Total RNA was isolated from left atrial tissues (*n* = 4 per group) by using TRIzol (Invitrogen, Carlsbad, CA, United States) from atriums according to the manufacturer’s instructions (Li et al., 2018). 15 μg of biotin-labeled complementary RNA were fractionated and hybridized to the Affymetrix Gene Chip and data were analyzed as previously described (Yang et al., 2010; Zhang et al., 2014; Dang et al., 2015). Gene expression profiling was performed using the mouse Genome 430 2.0 array according to the manufacturer’s protocols (Affymetrix, Inc., Santa Clara, CA, United States) (Zhang et al., 2014; Dang et al., 2015). The data of gene expression files are available at the Gene Expression Omnibus website under Accession No. GSE121516^1^. The intensity of gene expression was geometrically averaged and expressed as the fold ratio of Ang II-infused mice compared with the saline group for each time point. Bioinformatics analysis includes analysis of differential genes, gene ontology (GO), pathways, series test of clusters and gene co-expression networks, which were used to enrich the dataset for genes associated with AF as described previous (Zhang et al., 2014; Dang et al., 2015).

### Verification of Microarray Results by Quantitative Real-Time PCR Analysis

Total RNA was isolated from fresh heart tissues (*n* = 4 per group) using TRIzol reagent (Invitrogen, Carlsbad, CA, United States) according to the manufacturer’s instructions. The first strand cDNA was synthesized from 1 to 2 μg of total RNA by oligo (dT)-primed RT (iScript cDNA synthesis kit; Bio-Rad Laboratories). The mRNA levels of genes were determined by real-time PCR on an Applied Biosystems 7500 Real Time PCR System using SYBR Green (Applied Biosystems, Foster City, CA, United States). The cycling conditions consisted of an initial, single cycle of 4 min at 95°C, followed by 40 cycles of 10 s at 95°C, 45 s at 60°C, fluorescence acquisition, and then 95°C for 1 min, 60°C for 15 s. A melting curve was conducted from 60 to 95°C. Fluorescence was collected every 0.5°C. The levels of gene expressions were quantified relative to the level of GAPDH. The primers used in this study are listed in Supplementary Table 1.

### Statistical Analysis

Results are expressed as the mean ± standard error (SEM). Differences between groups were analyzed by non-parametric tests (Mann–Whitney or Kruskal–Wallis) or the parametric test one-way analysis of variance followed by the Tukey–Kramer test for group differences. *P*-value *of* < 0.05 was considered statistically significant.

## Results

### Ang II Increases BP and AF Incidence in Mice

**Figure 1:**
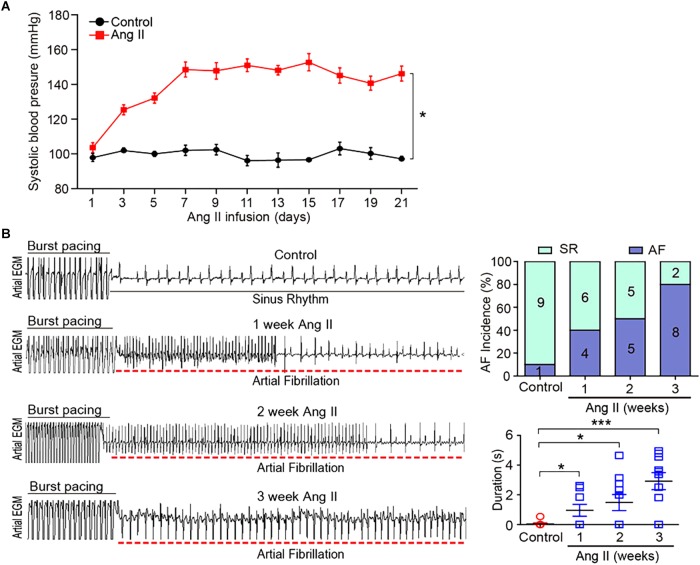
Ang II infusion induces elevation of blood pressure (BP) and AF susceptibility in mice. **(A)** Wild-type mice were injected with Ang II (2000 ng/kg/min) for 1, 2, and 3 weeks. Systolic BP was measured by the tail cuff method (*n* = 10 per group). **(B)** Representative AF incidence and duration were detected at weeks 1, 2, and 3 after Ang II infusion (left). Quantification of AF incidence and AF duration (right, *n* = 10 per group). Data are expressed as mean ± SEM. ^∗^*P* < 0.05; ^∗∗∗^*P* < 0.001 vs. saline.

To investigate the gene expression profiles in Ang II-induced AF, male wild-type mice were administered subcutaneous infusions of Ang II (2000 ng/kg/min) for 3 weeks. We found that systolic BP was progressively elevated at week 1, and remained high through week 3 of Ang II infusion (Figure 1A). Moreover, both incidence and duration of AF were progressively increased with increasing duration of Ang II infusion (Figure 1B).

### Ang II Increases Atrial Remodeling in Mice

**Figure 2:**
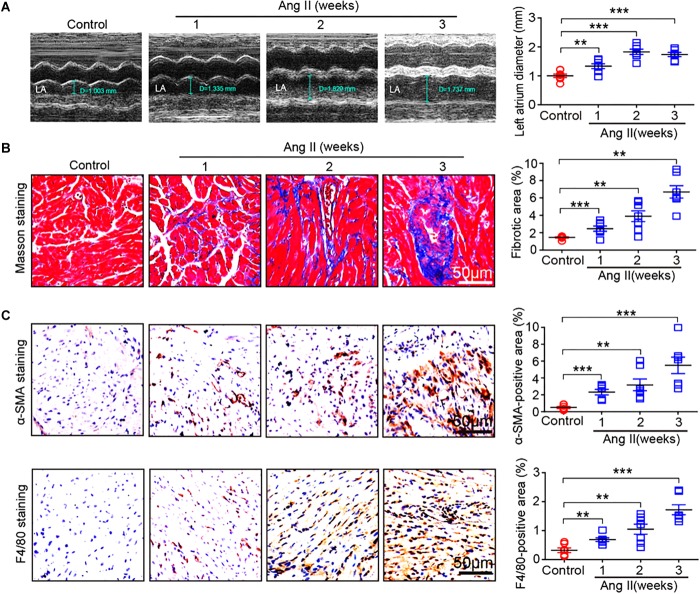
Ang II infusion promotes atrial remodeling. **(A)** Representative M-mode images of the left atrium at weeks 1, 2, and 3 after Ang II infusion. Quantification of atrial width (*n* = 10 per group). **(B)** Representative Masson trichrome staining (blue) of atrial sections, and quantification of fibrotic area (*n* = 8 per group). **(C)** Representative immunohistochemical staining of atrial sections with anti-α-SMA antibody (right), and quantification of α-SMA-positive fibroblasts (*n* = 8 per group). Representative immunohistochemical staining of atrial sections with anti-F4/80 antibody (right), and quantification of F4/80-positive macrophages (*n* = 8 per group). Scale bar: 50 μm. Data are expressed as mean ± SEM, and n represents the number of animals. ^∗∗^*P* < 0.01; ^∗∗∗^*P* < 0.001 vs. saline.

To determine the effect of Ang II infusion on atrial remodeling, we examined atrial structure and histological parameters. Echocardiographic measurement revealed that Ang II infusion time-dependently induced atrial enlargement (Figure 2A). Masson’s trichrome staining showed that atrial collagen deposition was significantly increased in a time-dependent manner (Figure 2B). Immunohistochemical staining further indicated that the numbers of α-SMA-positive fibroblasts and F4/80-positive macrophages were upregulated with increasing duration of Ang II infusion (Figure 2C).

### Analysis of Gene Ontology, Pathways, and Gene Expression Profiles in Ang II-Infused Atrium

**Figure 3:**
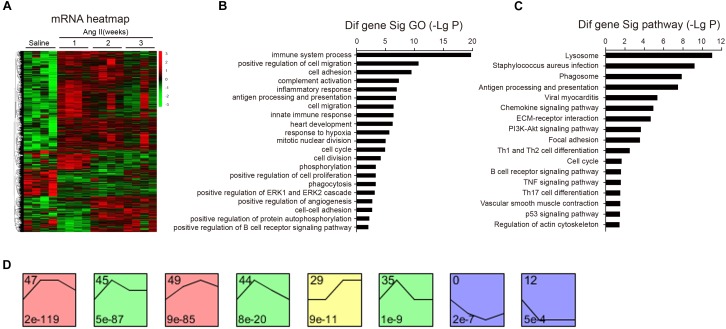
Analysis of gene ontology (GO) and KEGG pathways. **(A)** The heat map indicates the gene expression differences from the microarray between Ang II-infused atrium and saline control at weeks 1, 2, and 3 (*n* = 4 per group). The red color indicates up-regulation and green is for down-regulation. **(B)** GO analysis for differentially expressed genes. **(C)** Analysis of KEGG pathways for the differentially expressed genes in the atrium. LgP represents the logarithm of *P*-value. **(D)** The clustering analysis of differentially expressed genes in Ang II-infused atrium. Eight expression patterns (No. 47, 45, 49, 44, 29, 35, 0, and 12) of genes showed statistically significant difference (*P* < 0.00001) (colored boxes).

To identify the changes of gene expression in Ang II-infused atrial tissues, we performed time-series microarray analysis in left atrial tissues at weeks 1, 2, and 3 after Ang II infusion (*n* = 4 per time point). Microarray analysis showed that 1,719 genes were differentially expressed in Ang II-treated atrium at least at one time point compared to saline controls (*P* < 0.05). Of these, 1,283, 1,237, and 1,270 genes were markedly upregulated, whereas 436, 482, and 449 genes were downregulated at weeks 1, 2, and 3, respectively (Figure 3A).

We then identified biologically significant genes using GO and pathway analysis in Ang II-infused atrial samples. The most significant GO terms (*P* < 0.001) included immune system processes, positive regulation of cell migration, cell adhesion, complement activation, and the inflammatory response (Figure 3B). Moreover, we found that 87 pathways were significantly changed in Ang II-infused atrial tissues (*P* < 0.05), including lysosomal and phagosomal pathways and those involved in *Staphylococcus aureus* infection, antigen processing and presentation, viral myocarditis, chemokine and PI3K-AKT signaling, extracellular matrix (ECM)-receptor interaction, and focal adhesion (Figure 3C). Our results indicate that these GO terms and pathways may play critical roles in the initiation and development of AF induced by Ang II infusion.

**Figure 4:**
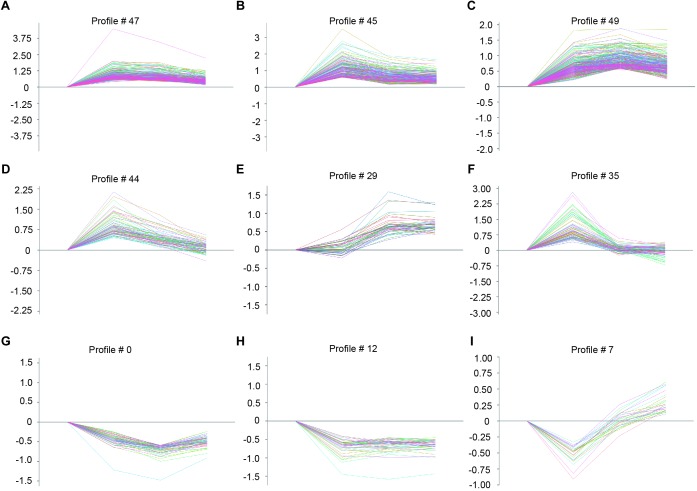
The time-series analysis of differentially expressed genes in each pattern **(A–I)**: Nos. 47, 45, 49, 44, 29, 35, 0, 12, and 7 (*n* = 4 pr group). The horizontal axis shows the time points, and the vertical axis shows the time series of gene expression levels.

To identify the gene expression patterns in the left atrium at weeks 1, 2, and 3 after Ang II infusion, we performed a hierarchical cluster analysis. In total, 1,719 differentially expressed genes were classified into 50 profiles in Ang II-infused atrial samples. Of these, eight profiles (No. 47, 45, 49, 44, 29, 35, 0, and 12) contained 1,157 genes and were significant (Figure 3D). The cluster analysis further revealed that the patterns of gene expression in profiles No. 47, 45, 44, and 35 were increased at week 1, and then gradually decreased at weeks 2 and 3. The expression levels of genes in profile No. 49 and 29 were progressively increased at weeks 1, 2, and 3, whereas the expression of genes in profile No. 0 was decreased at weeks 1 and 2, and increased at week 3. The expression of genes in profile No. 7 was decreased at week 1, and increased at weeks 2 and 3. The expression of genes in profile No. 12 was progressively decreased at all time points during Ang II infusion (Figure 4).

### Validation of Gene Expression

**Figure 5:**
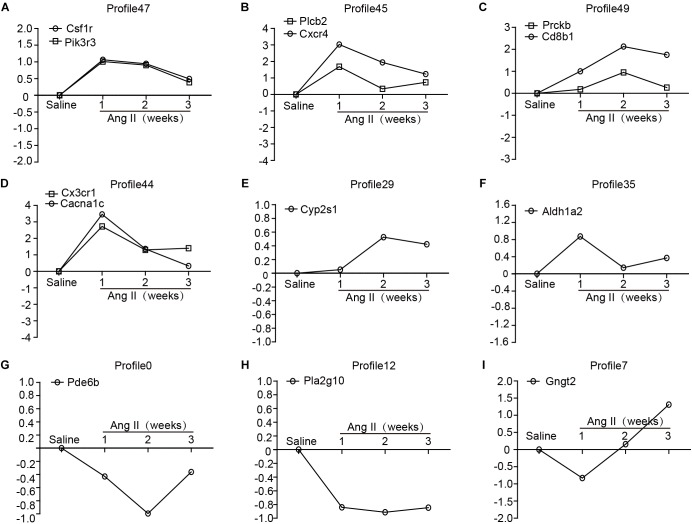
Validation of the results from microarray by qPCR analysis in Ang II-infused atrium at different time points. **(A–I)** The expression levels of selected 13 genes were verified by qPCR analysis (*n* = 4 per group), including Csf1r, Pik3r3, Plcb2, Cxcr4, Prckb, Cd8b1, Cx3cr1, Cacna1c, Cyp2s1, Aldh1a2, Pde6b, Pla2g10, and Gngt2. GAPDH as an internal control.

To further validate the microarray data, we performed quantitative polymerase chain reaction (qPCR) to analyze 13 gene expressions from eight significant and other non-significant profiles (Figure 5). As shown in Figure 5, the patterns of all gene expressions in No. 47 (Csf1r, Pik3r3), 49 (Prckb and Cd8b1), 45 (Plcb2 and Cxcr4), 44 (Cx3cr1 and Cacna1c), 35 (Aldh1a2), 29 (Cyp2s1), 0 (Pde6b), and 12 (Pla2g10) were similar to the results from the microarray analysis (Figure 5).

### Analysis of Gene Co-expression Network

**Figure 6:**
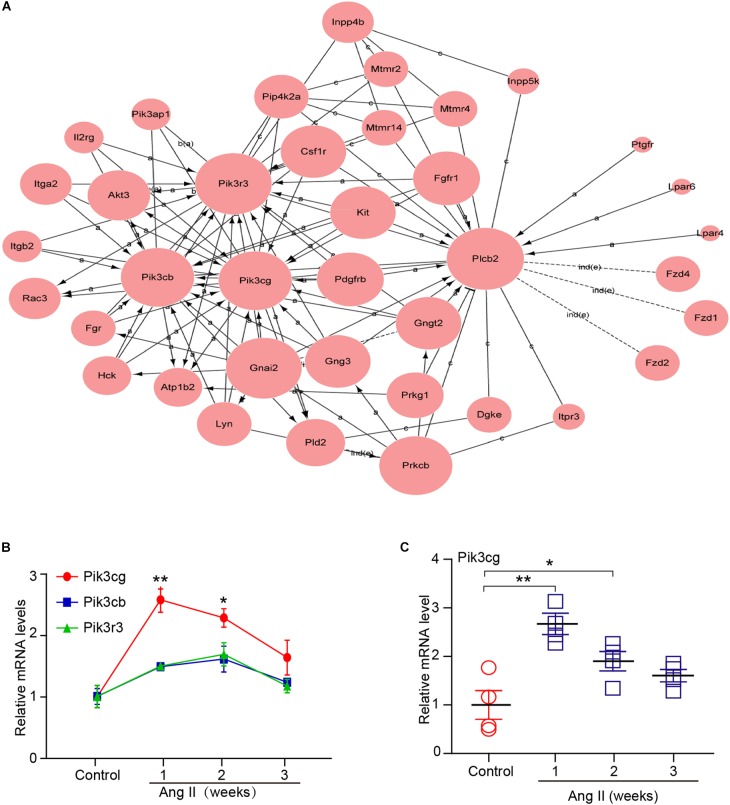
Analysis of gene co-expression network. **(A)** 37 genes selected from eight significant profiles (Nos. 47, 45, 49, 44, 29, 35, 0, and 12) were further analyzed by gene co-expression network with k-core algorithm. Cycle node shows genes, and edge between two nodes represents interaction between genes. **(B)** The expression of Pik3cg, Pik3cb, and Pik3r3 in the atrial tissues after Ang II infusion and saline control at weeks 1, 2, and 3 (*n* = 4 per group). **(C)** The mRNA level of Pik3cg was verified by qPCR analysis in Ang II-infused atrial tissues (*n* = 4 per group). Data are expressed as mean ± SEM, and n represents the number of animals. ^∗^*P* < 0.05; ^∗∗^*P* < 0.01 vs. control.

To determine which gene exerts a central role in AF induced by Ang II infusion, we further analyzed 37 selected genes in eight significant profiles using a gene co-expression network with a k-core algorithm based on the degree, k-core value, and betweenness centrality. Among these genes, Pik3cg (phosphatidylinositol-4,5-bisphosphate 3-kinase catalytic subunit gamma), which directly regulates 13 neighboring genes, had the highest degree and was at the center of the gene network (Figure 6A). Moreover, we found that three PI3K isoforms, including Pik3cg, Pik3cb and Pik3r3, were upregulated in Ang II-infused atrium at different time points, and the Pik3cg was the most highly expressed (Figure 6B). To further confirm the effect of Ang II infusion on Pik3cg expression, we performed qPCR analysis. Compared with saline control, Pik3cg mRNA level was increased at week 1, and then decreased at weeks 2 and 3 in Ang II-infused atrial tissues (Figure 6C). Together, these results suggest that Pik3cg may play a critical role in the regulation of AF.

## Discussion

In this study, we identified differentially expressed gene profiles, GO terms, and pathways during the development of AF. A total of 1,719 genes was found to be differentially expressed in Ang II-infused atrial tissues at weeks 1, 2, and 3, which are involved in multiple biological processes and signaling pathways. Importantly, Pik3cg was at the center of the gene co-expression network, and may be critical in the regulation of Ang II-induced AF.

Atrial fibrillation occurs in association with structural remodeling (i.e., fibrosis) and electrical remodeling (i.e., changes in ion channels) of the atria. The possible mechanisms for Ang II to trigger AF involve alterations in gene regulation and intracellular signaling pathways during AF (Griendling et al., 2000; Kumagai et al., 2003; Gao and Dudley, 2009; Gramley et al., 2010; Christophersen et al., 2017; Roselli et al., 2018). A recent study identified 163 independent risk variants at 111 loci and prioritized 165 candidate genes likely to be associated with AF. Many of them fall near genes where more deleterious mutations have been reported to cause serious heart defects and contractile dysfunction in mice or humans, such as Myh6, Nkx2-5, Pitx2, TBX5, Myh7, Pkp2, Sspn, and SGCA (Nielsen et al., 2018). Interestingly, mutations in a number of ion channel genes such as KCNQ1, KCND3 (Kv4.3), CACNB2,12, and CACNA2D4 have now been associated with AF (Chen et al., 2003; Weeke et al., 2014; Huang et al., 2017). To further define the gene alterations of AF, microarray analysis was performed to examine changes in gene expression profile in Ang II-induced cardiac remodeling and arterial hypertension (Dang et al., 2015; Zhang et al., 2018). We found that a total of 1,719 genes were found to be differentially expressed in the atrial tissues during the development of AF (Figure 3). Among them, Cx43 (also known as Gja1), Myh7, Csf1r, Pik3r3, Plcb2, Cxcr4, Cx3cr1, Cacna1c, Prckb, Cd8b1, and Cyp2s1 were significantly changed at different time points. Of note, several of these genes (Cx43, Myh7, CSF1R, CXCR4, and CACNA1) have been reported to be associated with AF, hypertension, inflammation, cardiac and vascular remodeling. For example, Cx43 plays a key role in electrical conduction velocity in cardiac tissues, and reduced expression of Cx43 was linked with AF (Shu et al., 2017). CSF1R (colony stimulating factor 1 receptor), a receptor for CSF1, was overexpressed in peripheral blood cells in hypertensive obese men compared with normotensive controls (Marteau et al., 2009). The chemokine receptor CXCR4 promoted hypoxia-induced pulmonary hypertension and vascular remodeling in rats, and inhibition of CXCR4 significantly attenuated these effects (Yu and Hales, 2011). CACNA1 is an alpha-1 subunit of voltage-dependent calcium channels, which mediates the influx of calcium ions into the cell upon membrane polarization. Both CACNA1A and CACNA1C may be significantly associated with BP change in the Han Chinese population (Hu et al., 2016). Together, these results indicate that gene expression alterations in the atrium induced by Ang II may be involved in AF inducibility.

Ang II is a key regulator of hypertension, inflammatory response and fibrosis through regulating multiple signaling pathways, such as NF-κB, NADPH oxidase, and TGF-β1/Smad (Cohn et al., 2000; Clarke et al., 2013). Fibrosis is a hallmark of atrial structural remodeling and enhances AF vulnerability through conduction abnormalities (Burstein and Nattel, 2008). Inflammation and reactive oxygen species can trigger AF and atrial fibrosis leading to the initiation and progression of AF (Gao and Dudley, 2009). Conversely, these effects are attenuated by anti-inflammatory agents, such as statins, corticosteroids, and angiotensin-converting enzyme inhibitors (Ozaydin, 2010). Consistent with these findings, our results showed that chronic Ang II infusion markedly induced elevation of BP, AF inducibility, left atrial dilation, fibrosis, and macrophage infiltration with increasing time (Figure 1, 2). Moreover, atrial fibrosis and inflammation-associated processes and signaling pathways were also time-dependently enhanced in Ang II-infused atrial tissues (Figure 3). Further, we identified that three PI3K isoforms, including Pik3cg, Pik3cb and Pik3r3, were upregulated in Ang II-infused atrium at different time points (Figure 6). PI3Ks have been classified into three major classes (I, II, and III) based on their structural features and functional similarities (Vanhaesebroeck et al., 2005). Class IA PI3Ks are heterodimeric lipid kinases composed of a catalytic subunit (p110α, p110β, or p110δ; encoded by PIK3CA, PIK3CB, and PIK3CD genes, respectively) and a regulatory subunit (p85). Class IB PI3Ks comprise a catalytic p110γ subunit (encoded by Pik3cg) and a regulatory p101 subunit (Vanhaesebroeck et al., 2005). PI3Ks generate lipids that regulate multiple intracellular signaling pathways (NADPH oxidase, AKT/eNOS, and TGF-β1/Smad) implicated in various cardiovascular diseases, including diabetes-induced cardiomyopathy and myocardial infarction (Kim et al., 2008; Pretorius et al., 2009; Lin et al., 2010). However, it is unclear which Pik3 isoforms are involved in the regulation of AF induced by Ang II. In this study, we found that Pik3cg was the most highly expressed and localized at the center of the gene network (Figure 5 and Supplementary Table 2), suggesting that Pik3cg may be critical in the development of AF induced by Ang II.

## Conclusion

We investigated Ang II-mediated molecular events associated with the development of AF using microarray analysis in a mouse model. A total of 1,719 genes was differentially expressed in Ang II infused atrium at different time points. These genes are involved in several biological functions. Among them, Pik3cg was localized at the center of the gene network, and may plays an important role in the development of AF. However, it will be important to determine the role of Pik3cg in Ang II-induced atrial remodeling and AF, and identify whether Pik3cg can provide a novel therapeutic target for AF.

## Ethics Statement

All procedures were approved by and performed in accordance with the Animal Care and Use Committee of Dalian Medical University. This study conformed to the Guide for the Care and Use of Laboratory Animals published by the U.S. National Institutes of Health.

## Author Contributions

Y-XW, XH, CC, L-XZ, and Z-CD conceived of the experiments, the acquisition of the data and analysis and interpreted the data. Y-XW participated in the statistical analysis of the primary data. H-HL and Y-LZ drafted the manuscript and provided funding to support the study. H-HL supervised the study. All authors approved the final version of the manuscript.

## Conflict of Interest Statement

The authors declare that the research was conducted in the absence of any commercial or financial relationships that could be construed as a potential conflict of interest.

## Footnotes

1 http://www.ncbi.nlm.nih.gov/geo/
