# Supplementary material for: Time Series Gene Expression Profiling and Temporal Regulatory Pathway Analysis of Angiotensin II Induced Atrial Fibrillation in Mice
Source: Front Physiol. 2019 May 29;10:597. doi: 10.3389/fphys.2019.00597 (PMC6548816; doi:10.3389/fphys.2019.00597)
Supplement: Supplementary file 1 [file Data_Sheet_1.PDF]

Table 1: primer sequences

Gene symbol	Forward primer (5’-3’)	Reverse primer (5’-3’)
Prkcb	TCAGAGCGGAAGGGTACAGA	TCGCTTGTCTCTAGCTTTTGGT
Cd8b1	ACTCAAGACGGCCCTTTCTC	GGAAGGACATCAACCACAGTCA
Pik3r3	ATCCAGCTGCGTAAGATCCG	CGTCTGCAACCACAGAACAA
Pik3cg	GCCCCGGGTAGGTCTAGATT	CATGCCCTATGCGACCTGA
Plcb2	CTGTCCCCTGGAGAGGTTTG	CCGGAGTTTCTGGCTCTGATT
Cxcr4	GAAACCTCTGAGGCGTTTGG	AAGTGTATATACTGATCGGTTCCAT
Cx3cr1	TTCCCATCTGCTCAGGACCTC	ACTAATGGTGACACCGTGCT
Cacna1c	CTCCTTTGGCATCCAGTCCA	TGAAGGCAATGAGCTTCAGG
Aldh1a2	TGTGGAGAAGGATGGATGCG	GAAAGCCAGCCTCCTTGATG
Cyp2s1	TCACGAGGACCACTTGCTTC	CGCTTACCTAAGGAGTAGGGC
Pla2g10	TGTGGTGAGTGCCCTATGC	TTTGTTCTCTGCTGGTCCACAA
Pde6b	GGAAATCCTGCCAACAAGGG	GCTCCAGCTCTGTACACTCC
Gngt2	TCCAGTGGTAAACAGCTCCC	AGACCTGGGTCCTGTTAGAG
Csf1r	GCCTCTTCCTCTGTTCCCTTTC	CCATTGCTCACACATCGCAG

Table 2: Fourteen genes identified by gene co-expression network with k-core algorithm

Gene symbol	Gene Title	Degree	Betweenness Centrality	K-core
Pik3cg	Phosphatidylinositol-4,5-Bisphosphate 3-Kinase Catalytic Subunit Gamma	16	0.010451885	5
Pik3r3	Phosphoinositide-3-Kinase Regulatory Subunit 3	15	0.012243528	5
Plcb2	Phospholipase C Beta 2	15	0.027754028	5
Cyp1a1	Cytochrome P450 Family 1 Subfamily A Member 1	15	0.00188758	8
Cxcr4	C-X-C Motif Chemokine Receptor 4	12	0.004471571	5
Cx3cr1	C-X3-C Motif Chemokine Receptor 1	12	0.001321393	5
Cxcr6	C-X-C Motif Chemokine Receptor 6	12	0.001321393	5
Ccr8	C-C Motif Chemokine Receptor 8	12	0.001321393	5
Ccr2	C-C Motif Chemokine Receptor 2	12	0.001421814	5
Gnai2	G Protein Subunit Alpha I2	11	0.023528574	5
Pdgfrb	Platelet Derived Growth Factor Receptor Beta	10	0.007005129	4
Fgfr1	Fibroblast Growth Factor Receptor 1	10	0.003882592	4
Cd8b1	CD8 Antigen, Beta Polypeptide 1	8	0.000303978	9
Prkcb	Protein Kinase C Beta	7	0.032289476	5
